# Policy Impact of the Rural Emergency Hospital Designation: A Mixed Methods Study of Leaders’ Conversion Experiences and Motivations

**DOI:** 10.1111/jrh.70240

**Published:** 2026-11-06

**Authors:** Christopher Wiggs, Dunc Williams, Kevin Wiley

**Affiliations:** ^1^ Department of Health Care of Leadership and Management, College of Health Professions Medical University of South Carolina Charleston South Carolina USA

**Keywords:** Medicare payment policy, mixed methods, Rural Emergency Hospital, rural hospital closure, rural health policy

## Abstract

**Purpose:**

The Rural Emergency Hospital (REH) designation, effective January 2023, offers financially distressed rural hospitals an alternative to closure to preserve emergency and outpatient services. Little is known about how hospital leaders weigh conversion or navigate its implementation. We characterized the pre‐conversion financial profile of converting hospitals and examined leaders' decision‐making and early implementation experiences.

**Methods:**

An explanatory sequential mixed‐methods study was utilized. Quantitative analyses drew on a hospital‐year panel (2013–2022) constructed from Medicare Cost Reports, the American Hospital Association Annual Survey, and the American Community Survey, comparing future REH converters with REH‐eligible and REH‐ineligible rural hospitals using one‐way ANOVA. Semi‐structured key informant interviews were conducted July through October 2025 with leaders representing 14 REHs, coded inductively by two investigators to thematic saturation.

**Findings:**

Before conversion, future REHs had lower days cash on hand, longer accounts receivable cycles, and higher uncompensated care than rural peers, reflecting a sustained multiyear margin decline. Leaders described conversion as a staged process: financial distress prompted consideration; a net‐feasibility analysis weighed the fixed facility payment and enhanced outpatient reimbursement against forgone inpatient, swing‐bed, and 340B revenue; external technical assistance and system affiliation enabled conversion; and implementation surfaced regulatory ambiguity, cross‐payer recognition gaps, payment delays, and the need to sustain community trust.

**Conclusions:**

REH conversion is a context‐dependent, multistage trade‐off rather than a universal solution. Earlier outreach before reserves are depleted, sustained technical assistance, and clearer regulatory and cross‐payer guidance may strengthen the model as a durable alternative to closure.

## 1 Introduction

Rural hospital closures remain among the most persistent barriers to healthcare access in the United States. Over the past two decades, more than 190 rural hospitals have closed, leaving millions of Americans in counties without nearby inpatient or emergency services. As of 2026, financial and operational instability continues to characterize the rural hospital landscape [1]. This instability has been worsened by large‐scale policy changes, including new administrative constraints on Medicaid and competition for limited resources [2]. Closures strain health systems, disrupt labor markets, and accelerate the demographic and economic decline of already vulnerable communities [2]. The Consolidated Appropriations Act (CAA) of 2021 established a new Medicare provider designation, the Rural Emergency Hospital (REH), effective January 1, 2023 [3, 4, 5].

The REH model offers eligible small rural hospitals and Critical Access Hospitals (CAHs) a pathway to avoid full closure while maintaining vital services [6, 7, 8]. To avoid discontinuing inpatient services, converting facilities receive an enhanced Medicare payment rate of 5% above standard outpatient rates and an annual facility payment intended to stabilize operations and preserve emergency and outpatient services [9, 10]. Hospitals qualify by maintaining a 24‐h emergency department compliant with federal staffing and service requirements [11, 12]. Currently, 54 hospitals nationwide have converted to REH status, serving disproportionately rural, low‐income, and medically underserved areas [5, 13, 14, 15].

Extant research has characterized hospitals most likely to pursue conversion [7, 16]. Eligible facilities tend to have low inpatient volumes, constrained payer mixes, and operating margins that make maintaining a traditional inpatient model financially untenable [2, 6, 7]. Pre‐conversion analyses confirm that converting hospitals were financially and operationally different from eligible non‐converters, exhibiting lower occupancy rates and revenues consistent with the REH model's financial logic [16, 17]. A growing literature has also examined the model's effects on access, community trust, and care quality [18, 19]. Some rural residents harbor skepticism about the program having expressed reluctance to use local REHs even when services remain available [17]. Workforce implications remain incompletely addressed, as federal staffing guidance for REHs conflicts with state nursing scope‐of‐practice requirements [20].

Despite this literature, little is known about how hospital leaders navigate conversion decisions. The factors that drive, constrain, or ultimately determine whether a hospital converts to REH status are multidimensional and extend beyond financial constraints. Organizations must simultaneously manage regulatory complexity, community relationships, service redesign, and strategic positioning, often under conditions of fiscal stress and uncertainty [21, 22]. Trade‐offs, such as loss of participation in the 340B drug pricing program, swing bed revenue, and disproportionate, share payments add further complexity [23, 24]. Prior literature has documented the characteristics of converting hospitals, but the process by which decision‐makers weigh priorities, engage stakeholders, and implement change remains poorly understood [25, 26].

This study seeks to address that gap using a mixed‐methods approach, including analysis of Medicare Cost Reports followed by an analysis of qualitative interviews of leaders who have undergone or are actively considering REH conversion. We draw on key informant interviews to develop a multi‐domain thematic analysis capturing the organizational, financial, regulatory, and community factors shaping conversion decisions and implementation [21, 26, 27, 28].

## 2 Methods

### 2.1 Study Design

We used an explanatory sequential mixed methods design to identify factors rural hospital leaders consider when deciding whether to convert to an REH, given the distinctive financial and contextual challenges their organizations face.

Quantitatively, we constructed hospital‐level panel using Medicare Cost Reports [29], the American Hospital Association Annual Survey (AHAAS) [30], and the American Community Survey (ACS) [31]. The study period covered years prior to REH conversion, since the first effective conversion date was January 1, 2023 [4]. The unit of analysis was the hospital‐year. Bivariate differences between treatment and comparison hospitals were tested using one‐way ANOVA for continuous variables with significance set a priori at *p* < 0.05. Data were winsorized at the 1% tails of each variable's distribution [32, 33]. Analysis was conducted using Stata 19.5.

We then employed a semi‐structured key informant interview protocol (Appendix 1) to capture the perspectives of REH leaders who had directly navigated the conversion decision and its aftermath. Open‐ended questions focused on the internal and external factors that shaped the decision‐making process, data and resources used, rationale for the final decision, and lessons learned. Interviews were conducted via Microsoft Teams, recorded with consent, transcribed, and analyzed using Delve.

We used an inductive coding approach [34]. The interview guide organized each conversation around four domains (internal and external factors shaping the decision, data and resources used, rationale for the final decision, and lessons learned), but no codes were specified in advance. Two authors independently open‐coded the first three transcripts, reconciled their codes into an initial codebook, and refined it iteratively, with disagreements resolved through consensus. Although the themes follow the guide's chronology, several, including system affiliation, payer recognition gaps, and the effect of community communication on emergency department volume, emerged from participants’ accounts rather than guide questions. Saturation was assessed as described under Study Sample generating a preliminary theme list and meeting routinely to compare interpretations and resolve any disagreements through consensus. Coding continued iteratively until saturation was achieved. Participant counts are reported to convey how widely each theme was shared, not to estimate prevalence.

### 2.2 Study Sample

The quantitative sample included all short‐term, general acute care rural hospitals in the United States that submitted cost reports for 2022, excluding hospitals with incomplete financial data (e.g., <360 reporting days) or not classified as general acute care. Rural hospitals were identified from the Federal Office of Rural Health Policy's (FORHP's) rural hospital list, applied retrospectively to each hospital's time series. We also classified as rural any hospital with Critical Access Hospital status, any hospital in the UNC Sheps rural closure file, and all Rural Emergency Hospitals [35]. Of 2130 rural hospitals evaluated for 2022, 421 were rural but REH‐ineligible, 1669 were rural and REH‐eligible, and 40 were future REH converters (four REH converters did not have complete‐year cost report data).

The qualitative component of this study included 12 interviews with leaders at participating REHs, representing approximately 32% (14/44) of all REHs nationwide when the study began. Recruitment followed a systematic, multi‐stage approach beginning with the UNC Sheps Center's list of REHs [15]. We sampled from the Rural Health Redesign Center (RHRC) [36], which promoted participation via email outreach and webinar announcements through its REH Technical Assistance Center. To give every facility equitable opportunity to participate, we built a contact database from facility websites, LinkedIn, and web‐scraped data using Python, and pursued outreach via email, LinkedIn, generic facility addresses, contact forms, and phone calls, with at least two contact attempts per facility. This yielded 13 completed interviews; one was excluded because the facility had reconverted to a non‐REH hospital, leaving a final analytic sample of 12 interviews representing 14 REHs. The REH population was small and fully enumerable; thus, we sought to recruit every operating REH rather than set a target sample size, and interviews concluded when outreach was exhausted in October 2025. Saturation was assessed concurrently. By the twelfth interviwew, no new codes emerged, consistent with evidence that saturation in homogeneous samples typically occurs within 9 to 17 interviews [37, 38].

## 3 Results

### 3.1 Quantitative Results

In 2022, relative to REH‐ineligible and REH‐eligible nonconverters, future REH converters reported less days cash on hand (DCOH) (82 vs. 108 and 144), higher days in net accounts receivable (A/R Days) (80 vs. 54 for both comparison groups), higher unreimbursed, uncompensated, and bad debt as a share of operating expenses (8.5% vs. 6.2% and 7.5%), and higher percent uninsured (10.1% vs. 8.7% and 9.0%) (Table 1). These statistically significant differences reflect generally weaker financial and operational performance among future REH converters; differences in capital purchases as a percent of total expenses were not significant.

**TABLE 1:** Unadjusted hospital characteristics by REH status, 2022.

	REH eligibility, all hospitals			
	Rural, not REH eligible	REH eligible, not REH	REH	*p*
*N*	421 (10.0%)	1669 (39.7%)	40 (1.0%)	
Days cash on hand	107.7 (111.3)	144.0 (112.7)	82.1 (88.8)	<0.001
Days in net accounts receivable	54.3 (29.7)	54.3 (38.0)	79.8 (78.6)	<0.001
Total unreimbursed, uncompensated, and bad debt (at cost) as a share of operating expenses (%)	6.2 (3.6)	7.5 (5.5)	8.6 (6.3)	<0.001
Percent uninsured	8.7 (4.6)	9.0 (5.4)	10.1 (4.5)	<0.001
Total capital purchases as a percent of total expenses (%)	5.6 (6.0)	6.1 (8.3)	6.4 (8.0)	0.506

*Note*: Percent uninsured was derived from ACS data and reflects the Zip Code Tabulation Area (ZCTA) for the hospital.

Trend data from 2013–2022 (Figure 1) show future REH converters consistently averaging lower days cash on hand and higher accounts receivable days and uncompensated care burden than eligible and ineligible rural peers. Lower cash reserves signal reduced capacity to absorb shocks; higher A/R days reflect operational revenue cycle inefficiency; and higher uncompensated care reflects lower effective reimbursements. Together, these trends show a multi‐year pattern of worsening financial performance preceding REH conversion.

**FIGURE 1:**
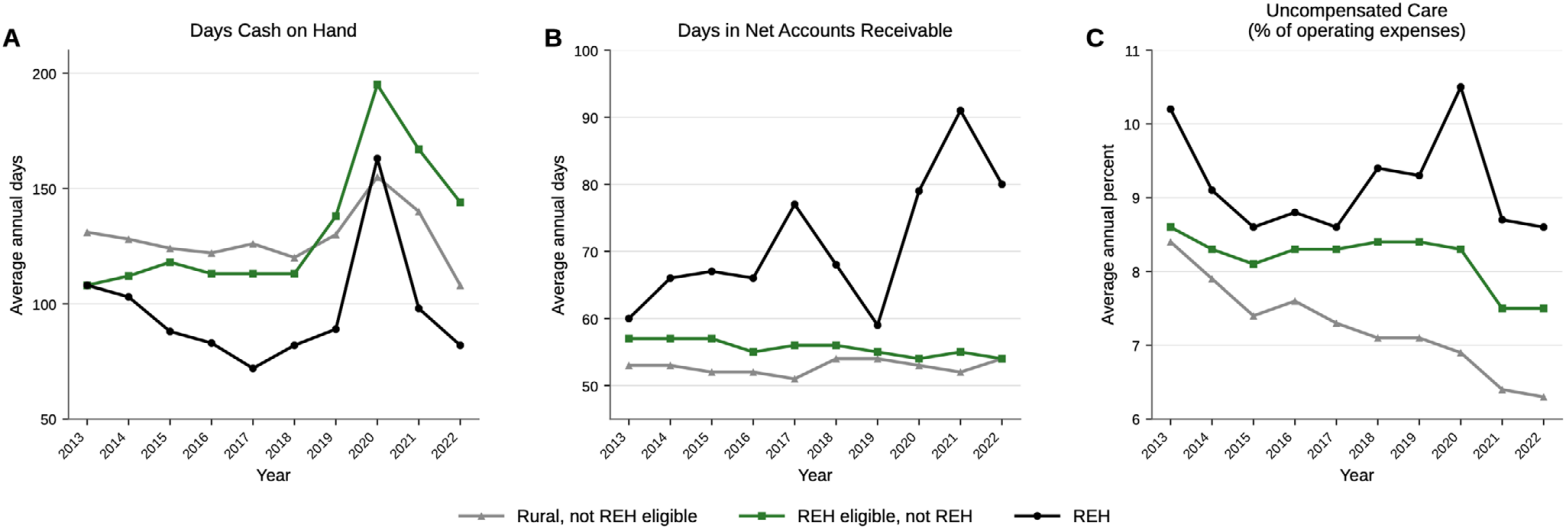
Financial trends among future REH converters, eligible nonconverters, and non‐eligible rural hospitals, 2013‐2022.

### 3.2 Qualitative Results

Twelve interviews were conducted between July and October 2025 with participants representing 14 REHs (one participant held responsibility across multiple facilities). Participant and facility characteristic are summarized in Table 2. Conversion dates ranged from February 2023 to March 2026 with 13 facilities already operating as REHs and one in mid‐conversion. Analysis yielded 7 themes describing the conversion process, presented in sequence: financial distress prompted consideration of the model; financial feasibility shaped the final decision; external support and system affiliation enabled conversion; service‐line redesign reflected local adaptation; regulatory ambiguity and payment delays complicated implementation; and community perception shaped whether trust was maintained. The full set of themes and subthemes, along with representative participant quotes, are summarized in Table 3.

**TABLE 2:** Participant and facility characteristics (12 interviews representing 14 rural emergency hospitals).

Interviews conducted^a^	12	REHs represented^b^, ^c^	14
**Participant role (*n* = 12)**		**Facility characteristics (*n* = 14)**	
Chief executive officer	8	States represented	11
System president	1	System‐affiliated	7
Board president/interim CEO	1	Independent	7
REH hospital administrator	1	*Prior Medicare designation*	
Assistant administrator	1	Prospective payment system (PPS)	6
		Critical access hospital (CAH)	5
		Sole community hospital (SCH)	2
		Medicare‐dependent hospital (MDH)	1
		*REH status at interview*	
		Operating as an REH	13
		Completing conversion	1

Abbreviations: CAH, critical access hospital; CEO, chief executive officer; MDH, Medicare‐dependent hospital; PPS, prospective payment system; REH, rural emergency hospital; SCH, sole community hospital.

^a^ Interviews were conducted July–October 2025. One participant held executive responsibility across multiple affiliated facilities, accounting for the difference between the 12 interviews and 14 REHs represented.

^b^ Facilities were located in 11 states: Alabama, Arkansas, Georgia, Idaho, Kansas, Kentucky, Michigan, Mississippi, Missouri, New Mexico, and New York.

^c^ Official REH conversion dates ranged from February 2023 to March 2026.

**TABLE 3:** REH conversion key informant interview themes and subthemes (*N* = 12).

Theme	Subtheme	*N*	Representative quote
Experiencing financial distress and declining inpatient viability	**Overall theme**	**12**	
	Decreasing profitability/liquidity with increasing costs	12	“In late 2023 and into 2024, we were losing a tremendous amount of money. It was not sustainable…several million a year…so that really drove the decision to do something.”
	Low inpatient volume/census	9	“In 2023, we experienced about a $1.5 million loss…our inpatient volumes started to decline significantly…We went from about 36 discharges every month to about 11.”
	Decreasing reimbursement	6	“We got to a place financially where we were needing some immediate assistance…our low volume adjustment actually stopped…we were at one point owed $350,000 by Medicare that was back pay.”
Weighing net financial feasibility before choosing REH conversion	**Overall theme**	**12**	
	Weighing the net financial tradeoffs of conversion	12	“Take your financials from the previous two or three years…you're going to have OPPS plus 5%…put all the tradeoffs in…run the calculations and have two people do it.”
	Context‐dependent fit of the REH model	8	“REH can't be the solution in totality…adding $3.5 million doesn't make up for that gap…some have a lot of 340B and swing bed revenue that's more than $3.5 million.”
Leveraging external support and system affiliation to facilitate conversion	**Overall theme**	**10**	
	Technical assistance and consultant support	9	“I've been appreciative for…the technical assistance [RHRC] provided…helping me as a hospital CEO to see we're a pioneer leading this REH and not a Guinea pig.”
	State, hospital association, and federal government support	7	“The licensing opportunity was not available for us in the state of Missouri. So, I engaged with our state legislators…our bill…became the whole healthcare omnibus bill for the entire legislative season.”
	Peer learning	5	“I felt it was really important that I personally interview every CEO of a hospital that converted…I listed all twenty‐nine of them…and brought my report of that due diligence to the board.”
	Health system & clinical partnerships	4	“Being part of a system made our decision so much easier…when we made the decision…I could tell them you've got a job at our main hospital.”
Repositioning the rural hospital service model through conversion	**Overall theme**	**12**	
	Right‐size services to community needs	10	“Maybe we should embrace this opportunity and get rid of inpatient medicine entirely. Focus on what we can do well…emergency care, family practice and physical therapy.”
	REH framed as preserving access to care	6	“The financial aspect was key, but also…it allowed us to remain in that community…that desire to stay in the rural community to provide care but be financially stable.”
	Strategic service‐line repositioning after conversion	8	“We only ever had six inpatient rooms…one we converted to additional physical therapy space…three rooms we use for a visiting specialist…a cardiologist, an orthopedist, a urologist.”
Navigating regulatory ambiguity during conversion	**Overall theme**	**10**	
	Payer recognition uncertainty	7	“How states pay these REHs for their Medicaid patients is a little bit problematic because it's not codified in the CMS regulation. They left Medicaid out.”
	State licensure & legislation	5	“The state did not recognize REH as a hospital. We lost the ability to get upper payment limit funds…disproportionate share…some years $4 million less per year.”
Managing administrative and payment delays during conversion	**Overall theme**	**8**	
	Delayed payments and cash‐flow bridge	7	“When you change CCNs, everything tied to the CCN also must change…the entire billing system had to be revamped. We held all our claims from January 1 until March 19th.”
	REH application and approval delays	7	“It took a solid year from the time that we had our paperwork in to the time we got designation.”
Managing community perception and trust before, during, and after conversion	**Overall theme**	**11**	
	Communication failures & their consequences	8	“The community is upset. [It] was not rolled out good from the beginning…they were not kept in the loop…now we're seeing maybe 10 or 12 [patients].”
	Reframing conversion as preservation, not loss	7	“They didn't feel like they were losing a hospital…they said you shouldn't talk about losing anything. You should simply say you are focusing on emergency medicine.”
	Proactive community engagement	9	“I was on the road probably for two solid years, like pre‐conversion, during conversion, post‐conversion, just communicating with the community at town hall meetings.”

Theme 1: Experiencing financial distress and declining inpatient viability

All 12 participants situated the conversion decision within deteriorating finances that made the inpatient model difficult to sustain, citing persistent operating losses, thin cash reserves, and rising labor, supply, and overhead costs, consistent with quantitative evidence of weaker margins among REH‐eligible hospitals [6]. For example, one participant stated, “From the middle of 2022 till the middle of 2024, our net revenue increased 12% and our total operating expenses increased 39% giving a 27% negative impact on our operating margin…expenses were running away with us.” Most (75%) cited low inpatient volume and census compounding pressures and half described declining or inadequate reimbursement, including unfavorable payer mix, growing Medicare Advantage penetration eroding low‐volume payment adjustments, and delayed federal support.

Theme 2: Weighing net financial feasibility before choosing REH conversion

Nearly all participants (92%) described a deliberate financial feasibility analysis before converting, weighing the predictable facility payment and enhanced outpatient reimbursement (OPPS plus 5%) against forgone inpatient revenue and programs (see Table 4), typically modeling the net effect using 2 to 3 years of hospital‐specific cost report data with external partners. Two‐thirds stressed that conversion is not universally appropriate and that net benefit depends on inpatient volume, service and payer mix, outpatient potential, and whether the facility payment offsets anticipated losses [10, 24, 39]. As one participant explained,

REH is not financially a cakewalk that you can just take the facility payment and not provide any services because for most hospitals it's not going to be sufficient to cover all your expenses. It works well with a hospital that's operationally active, meeting the needs of the community and still bringing in some patient revenue.

Theme 3: Leveraging external support and system affiliation to facilitate conversion

**TABLE 4:** Factors weighed in assessing net financial feasibility of REH conversion.

Factor weighed	Weighs toward conversion	Weighs against conversion	Context that determines which dominates
**Fixed monthly facility payment (∼$3.6 M/year)**	Predictable, volume‐independent revenue improves stability and liquidity; offsets inpatient losses at low‐volume facilities	A flat sum; where annual losses exceed it (e.g., a $5–8 M gap), it does not close the shortfall	Magnitude of current operating losses relative to the payment; inpatient volume
**Outpatient rate change (OPPS + 5%)**	Raises outpatient Medicare payment for prospective payment system (PPS)–based hospitals	For cost‐reimbursed critical access hospitals (CAHs), can fall below prior cost‐based reimbursement, lowering revenue per lab, visit, and outpatient service	Prior Medicare payment basis (PPS vs. cost‐reimbursed CAH); outpatient share of revenue
**Inpatient census/volume**	Shedding chronically low, money‐losing inpatient care removes a persistent loss and staffing burden	Forgoes all inpatient revenue; poor fit where daily census is still meaningful	Average daily census; proportion of revenue derived from inpatient care
**Swing‐bed/post‐acute revenue**	Minimal downside where swing‐bed use is low; services can shift to a distinct‐part skilled nursing facility	Material loss where swing‐bed revenue is a significant margin source	Reliance on swing‐bed revenue; availability of a distinct‐part SNF alternative
**340 B drug pricing**	Negligible impact where 340 B savings are small	REHs lose 340 B eligibility; facilities with large 340 B revenue may lose more than the facility payment adds	Size of 340 B drug savings/revenue
**Current financial trajectory**	For facilities near insolvency, conversion provides a viable path to stay open	For financially stable facilities, adds disruption with limited financial upside	Proximity to insolvency or closure; available cash reserves

*Note*: Factors reflect considerations raised by participants in this study. Program payment parameters (the fixed monthly facility payment, the OPPS + 5% outpatient enhancement, loss of 340 B eligibility, and cost‐based reimbursement for CAHs) are drawn from sources already cited in this manuscript [36, 39, 48].

Ten of the 12 participants described external support, particularly the Rural Health Redesign Center (RHRC) and its REH Technical Assistance Center, cited by nine, as important for interpreting regulations, modeling financial impacts, and reducing uncertainty. Illustrating this one leader said, “[RHRC] gave us a great toolbox that if you're going to make this transition, here's all the steps you need to take…[the] permissions you need to get from regulators…the communication we need to do in the community.” Seven described how state agencies, hospital associations, legislators, and CMS intermediaries shaped the ease of conversion, sometimes through direct legislative lobbying action. Informal peer support facilitated in part by RHRC, was cited by 41.7% as helpful for validating assumptions against early converters. Four of 12 participants credited system affiliation with easing the operational and financial burden of conversion, a dimension underexamined in prior facility‐level REH literature [5, 6, 39, 40].

Theme 4: Repositioning the rural hospital service model through conversion

For all participants, conversion functioned as a service‐line redesign rather than a purely financial maneuver. Ten of 12 right‐sized services to community needs, concentrating resources on 24/7 emergency care, primary care, laboratory and imaging, and specialty clinics, and 8 of 12 repositioned space, staffing, and service lines post‐conversion. One participant elaborated, “We couldn't continue to sustain financial losses; we would have closed. So, this [conversion] gave us an opportunity to focus on our outpatient service lines, 24/7 emergency department, which is vital to the community.” Several leaders used conversion to expand rather than contract their footprint, adding wound care, reopening geriatric‐psychiatry lines, and building new emergency departments with observation beds. Six of 12 participants framed conversion as preserving a meaningful long‐term local care presence and local employment.

Theme 5: Navigating ambiguity during conversion

Ten of the 12 participants described regulatory ambiguity and inconsistency, most often over whether Medicaid, Tricare, and other payers would recognize REHs as hospitals and reimburse them accordingly. This was captured by a participant who noted, “In the state of Alabama, you do not get paid by Medicaid if you're a REH…in the federal regulations of how it was written, it only included Medicare, it didn't specifically say Medicaid.” Five of 12 pointed to gaps in state licensure and legislation, which created barriers when statutes, supplemental payment programs, or grant language had not yet been updated to recognize the designation.

Theme 6: Managing administrative and payment delays during conversion

Eight of 12 participants experienced administrative lag and cash‐flow strain during the transition to REH status. One participant noted, “From January 1 to March, we were holding bills. So now you're talking about cash flow challenges. I'm fortunate to be part of a bigger system but it's still a drain on the system not to have the cash flow for the organization.” Seven described delays in facility payments, Medicare reimbursement, payer billing, and provider enrollment, often tied to CMS Certification Number changes and the resulting cascade of billing‐system updates. An equal number reported application and approval delays from layered reviews by Medicare Administrative Contractors, states, regions, and the federal government, and effective‐date uncertainty.

Theme 7: Managing community perception and trust before, during, and after conversion

Eleven of 12 participants emphasized managing community perception, since residents may interpret the loss of inpatient beds as an outright closure. Eight of 12 described how faltering communication eroded community trust resulting in lower emergency department volumes. To address this, 9 of 12 leaders engaged proactively through town halls, radio, social media, newspapers, and civic outreach, and 7 of 12 worked to reframe the REH conversion as a preservation of care rather than a loss. One participant captured how these elements fit together:

You got to get everybody on board… understanding where you are and where you're trying to go, and the communication of that to staff, to the community, to the board of directors… because there's a lot of misinformation… [Competitors] will say, well, [our hospital] is converting to just an emergency room only. Well, that's not the case… so communication is probably the biggest component… we're making decisions for the long‐term viability of being able to provide healthcare for the community.

Across the seven themes, conversion emerged less as a single decision than as a sequence of interacting pressures and responses, beginning with the financial difficulties that trigger conversion and extending to community management participants viewed as central to long‐term success.

## 4 Discussion

This mixed‐methods study combined national cost report data on 44 Rural Emergency Hospitals (REHs) operating in 2022 with 12 key informant interviews representing 14 hospitals to characterize how rural hospital leaders decide to convert and their experiences implementing the model. Quantitatively, REHs entered 2022 as a financially distinct subset of the rural hospital landscape, with lower margins than urban, rural REH‐ineligible, and REH‐eligible non‐converting hospitals, consistent with prior REH studies [17, 41, 42]. These reflect a persistent, multi‐year trajectory of margin erosion predating the designation's availability. Our qualitative findings expand this by showing how leaders weigh fiscal needs, organizational capacity, regulatory uncertainty, and community trust together.

Respondents characterized REH conversion as a response to sustained financial distress rather than the model's intrinsic design features [3, 4]. Persistent operating losses, limited liquidity, and escalating labor costs shifted organizational strategy from maintaining inpatient services toward identifying alternatives to closure, which is consistent with prior evidence that rural hospital closures are most strongly associated with prolonged negative margins, low patient volume, and constrained access to capital [7, 43, 44]. Our analyses corroborate these studies. Although the REH designation was designed as a voluntary alternative to closure under the CAA [4, 5], hospitals typically reach conversion consideration after the inpatient model has become financially untenable, following a prolonged structural decline.

Liquidity constraints further distinguish converters [44]. Days cash on hand among REHs was substantially lower than among both REH‐eligible non‐converting hospitals and other rural hospitals, indicating reduced capacity to absorb shocks or sustain operations during transition, and is supportive of liquidity as a key predictor of rural hospital vulnerability and closure risk [44, 45, 46]. The REH model may thus function less as a proactive redesign and more as a late‐stage intervention for financially distressed facilities with limited remaining options [5, 17, 22, 27].

Decision‐making centered on evaluating the fixed facility payment and enhanced outpatient reimbursement against forgone inpatient, swing bed, and 340B revenue [16]. These trade‐offs represent the broader literature emphasizing payer mix, service line composition, and scale in determining financial viability [5, 17, 18, 22, 47]. At scale, modest reimbursement changes can generate large proportional effects on margins, echoing participants’ emphasis on inpatient volume, outpatient services, and payer mix as central determinants of feasibility. Converting hospitals exhibited the lowest commercial‐to‐Medicare payment ratios among all groups, limiting their capacity to offset public payer shortfalls through commercial revenue. Hospitals serving more Medicare and Medicaid patients face greater strain and less flexibility under payment reforms [17, 18, 48]. These results indicate that REH conversion is a context‐dependent trade‐off, and that size, payer mix, and pre‐conversion financial position may help identify facilities most likely to benefit.

Participants also emphasized technical assistance in navigating a novel, operationally complex designation, with the Rural Health Redesign Center's Technical Assistance Center frequently cited as critical for financial modeling, regulatory interpretation, and implementation planning. Rural hospitals, particularly independent facilities, often lack the administrative and analytical capacity to respond to major policy changes [18, 49]. REHs and REH‐eligible hospitals were less likely to be system‐affiliated than other hospitals, suggesting many lack the infrastructure and economies of scale membership can provide.

Independent hospitals in geographically isolated or resource‐constrained settings may face greater barriers to conversion. REHs were disproportionately government‐owned and less often not‐for‐profit. Most financially vulnerable rural hospitals are more likely to be publicly owned and to have limited access to private capital [6, 17], suggesting technical assistance availability may shape both conversion and post‐conversion performance.

Participants described additional implementation challenges including adapting service REH requirements, navigating regulatory ambiguity, managing payment delays, and addressing community perceptions. REHs entered conversion with longer accounts receivable cycles and higher levels of uncompensated care than other hospital groups, patterns consistent with administrative complexity and revenue‐cycle disruption during the transition and delays observed in other rural health policy transitions [50].

Community response was an ongoing consideration. Participants described initial skepticism about the loss of inpatient services but stressed that sustained engagement and communication helped maintain trust and utilization. Rural hospitals serve as community anchors and the sensitivity of access perceptions may affect downstream healthcare outcomes [51]. Implementation thus represents a distinct, consequential phase with implications for both financial performance and community relationships. The consequences of this phase are evident in early exits from the model: as of July 2026, 2 of the 56 hospitals that had converted to REH status had closed and 3 no longer participated in the model [15]. Within our study, one completed interview was excluded because the facility had already reconverted to a non‐REH hospital, and one participating REH closed after interviews concluded. These exits are consistent with our findings that converters entered the designation with thin cash reserves and that lapses in community communication preceded declining emergency department use. Together, they suggest the facility payment can stabilize operations but cannot by itself offset sustained volume loss or deep pre‐existing deficits. Policies strengthening the REH model may benefit from greater post‐conversion support, including clearer regulatory guidance, improved payment infrastructure, and community engagement resources.

These findings carry several policy implications. First, because hospitals appear to reach the REH decision after years of eroding margins, earlier and more proactive outreach to REH‐eligible hospitals, before reserves are depleted, could preserve more options and reduce the risk of converting too late to stabilize. Second, the centrality of external technical assistance and REHs’ lower rates of system affiliation, sustained federal or state investment in organizations like the Rural Health Redesign Center is a core structural complement to the model, particularly for hospitals lacking system‐level capacity. Third, the substantial differences in size, payer mix, and liquidity between REHs and other REH‐eligible hospitals argue against treating conversion as a default recommendation for all distressed rural hospitals; decision‐support tools incorporating hospital size, commercial‐to‐Medicare payer ratio, and cash reserves may help determine when and how conversions occur.

Future research should analyze post‐conversion staffing and financial outcomes over longer periods, test whether pre‐conversion size, payer mix, and liquidity predict which REHs stabilize, and link leaders' feasibility assumptions to actual performance. Additional qualitative research with hospitals that rejected conversion, or later reconverted, would clarify when the REH model suits closure prevention.

### 4.1 Limitations

This study has several limitations. Medicare Cost Reports are self‐reported and can exhibit inconsistencies [52], though their availability across CMS‐reimbursed providers makes them a commonly accepted source [52]. The qualitative sample captured perspectives from leaders at 14 of 44 REHs operating at the time of data collection; because rural hospitals operate within diverse, highly localized financial and community contexts, factors shaping conversion decisions may differ across settings not fully captured here. Finally, half of the REHs in this study were system‐affiliated; system‐level resources and infrastructure may shape conversion decision‐making divergent from independent rural hospitals, which typically have fewer resources and face more acute financial and operational pressures. Because we sampled operating REHs, we could not characterize the decision‐making or post‐conversion experience of hospitals that later closed or left the model.

## 5 Conclusion

Combining nationally representative financial and operational data with key informant interviews, we found that REHs entered conversion as a financially distinct and consistently declining subset of the rural hospital landscape, with substantially lower overall margins than other REH‐eligible hospitals and average net revenue roughly one‐third that of their non‐converting peers. REH conversion is best understood as a multi‐step process beginning with years of shrinking margins, followed by a careful feasibility analysis influenced by hospital size and payer mix, and ongoing implementation challenges related to regulatory uncertainty and community trust. Policymakers and rural health technical assistance organizations seeking to strengthen the REH model as an alternative to closure should systematically address challenges at each stage of this process, including financial trade‐offs to ensure the model functions as a lasting safety valve for the rural hospitals most at risk of closing.

## Funding

The authors received no financial support for the research, authorship, and publication of this article.

## Conflicts of Interest

The authors declare no conflicts of interest.

## Supporting information




**Supporting File 01**: jrh70240‐sup‐0001‐SuppMat.docx
