# Supplementary material for: Policy Impact of the Rural Emergency Hospital Designation: A Mixed Methods Study of Leaders’ Conversion Experiences and Motivations
Source: J Rural Health. 2026 Nov 6;42(4):e70240. doi: 10.1111/jrh.70240 (PMC13742113; doi:10.1111/jrh.70240)
Supplement: Supplementary file 1 [file JRH-42-0-s001.docx]

Appendix 1: Semi-Structured Key Informant Interview Protocol Questions

1. What factors or challenges (both internal and external) prompted your organization to explore the rural emergency hospital designation?
2. Describe the organization’s decision-making process when considering whether to convert to the rural emergency hospital designation.
   a. What were the three most important factors that affected the decision-making process?
   b. What data or information were vital in the decision-making process?
   c. What tools and resources did you utilize in the decision-making process?
   d. Why did [ORGANIZATION] ultimately choose to move forward (or not move forward) with converting to the rural emergency hospital designation?
3. What key lessons would you share with rural hospital executives who are assessing whether converting to a Rural Emergency Hospital designation is the best strategy to address their organization's challenges?
4. Before we conclude the interview, is there anything else that you feel is important related to our topic that we did not discuss ?
